# Breaking through a phylogenetic impasse: a pair of associated archaea might have played host in the endosymbiotic origin of eukaryotes

**DOI:** 10.1186/2045-3701-2-29

**Published:** 2012-08-22

**Authors:** James S Godde

**Affiliations:** 1 Department of Biology, Monmouth College, 700 East Broadway, Monmouth, IL 61430, USA

**Keywords:** Eukaryogenesis, Endosymbiosis, Phylogeny, Thermophiles

## Abstract

For over a century, the origin of eukaryotes has been a topic of intense debate among scientists. Although it has become widely accepted that organelles such as the mitochondria and chloroplasts arose via endosymbiosis, the origin of the eukaryotic nucleus remains enigmatic. Numerous models for the origin of the nucleus have been proposed over the years, many of which use endosymbiosis to explain its existence. Proposals of microbes whose ancestors may have served as either a host or a guest in various endosymbiotic scenarios abound, none of which have been able to sufficiently incorporate the cell biological as well as phylogenetic data which links these organisms to the nucleus. While it is generally agreed that eukaryotic nuclei share more features in common with archaea rather than with bacteria, different studies have identified either one or the other of the two major groups of archaea as potential ancestors, leading to somewhat of a stalemate. This paper seeks to resolve this impasse by presenting evidence that not just one, but a pair of archaea might have served as host to the bacterial ancestor of the mitochondria. This pair may have consisted of ancestors of both *Ignicoccus hospitalis* as well as its ectosymbiont/ectoparasite ‘*Nanoarchaeum equitans’*.

## Introduction

Eukaryotic genomes are chimeric in nature, in that they appear to contain genes contributed over time by all three domains of life. A recent analysis of the yeast *Saccharomyces cerevisiae* genome estimated that approximately 30% of its genes had originated from Bacteria, and about half that number appeared to be eukaryotic innovations, while only around 7% could be linked to archaeal origins [1]. Despite their smaller number, however, the latter category was found to be more essential to viability, more highly expressed, and more connected to protein interaction networks [1]. In general, archaeal-derived genes can be thought of as largely “informational”, while bacterial-derived genes tend to be more “operational”, in that they typically are associated with certain aspects of metabolism [2]. When the human genome is analyzed in a similar fashion, the percent of innovative genes without detectable prokaryotic homologs quadruples to nearly 60%, as might be expected from our increased level of complexity, while the relative proportions of putative bacterial- to archaeal-derived genes becomes even more skewed (36% to 4%, respectively) [3]. Nonetheless, the dichotomy in function as well as overall importance to the cell is still maintained [3]. This leads to a logical question- where did all the prokaryotic-derived genes come from? Evolutionary biologists typically point to horizontal gene transfer and/or endosymbiosis to explain the initial appearance of these genes in eukaryotes. These differ in that the former method is exceedingly common among prokaryotic cells while the latter is so vanishingly rare that it is thought to have occurred only a few times in the 1 to 2 billion years since the last eukaryotic common ancestor (LECA) arose. The notion that mitochondria arose via endosymbiosis of an α-proteobacterial guest has received wide acceptance among biologists [4]. This would suggest that some combination of endosymbiosis and horizontal transfer accounts for eukaryotic genes of bacterial origin. What has changed rather significantly, however, is the estimated timing of this endosymbiotic event. While it was originally hypothesized that mitochondria arose early enough in eukaryotic evolution so that some protists which lacked this organelle originated before this event, subsequent molecular analysis supports the view that amitochondriate eukaryotes have instead resulted from a loss of this organelle [5]. The absence of extant nucleated cells which never hosted mitochondrial-type organelles has therefore moved this endosymbiotic event closer to the appearance of the LECA [6,7]. One topic that remains the subject of sharp dispute is the origination of the nucleus and the associated appearance of archaeal-derived genes.

### Origin of the nucleus

**Figure 1:**
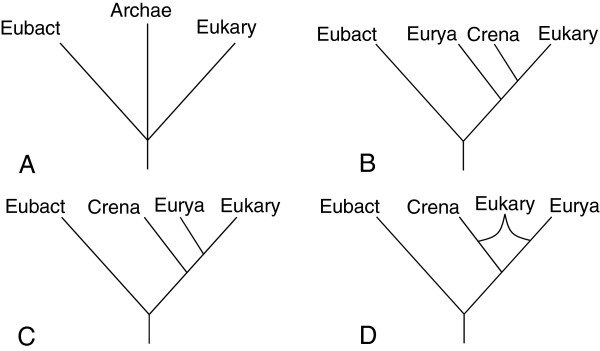
**Different phylogenetic trees describing the relationship between eukaryotes and archaea.A**) Three Sisters Tree- the three domains of life are nearly equally related, with no special relationship between Eukarya and Archaea. Although a trivergence is not allowed in phylogeny, one is shown here since it would be unclear in this model which domain diverged from the others first. **B**) Crenote Tree- (aka Eocyte Tree) Eukarya and Crenarchaeota are sister groups. **C**) Euryote Tree- Eukarya and Euryarchaeota are sister groups. **D**) Synote Tree- Eukarya are derived from a fusion between Crenarchaeota and Euryarchaeota. For simplicity, the genetic contributions arising from the α-proteobacteria endosymbiont are not included in any of these trees.

The theory that the nucleus also arose via endosymbiosis was first proposed in a classic 1905 paper by Mereschkowsky [8]. There, in an insight that was many decades ahead of its time, the author proposed that a bacterium was “invaded by small micrococci which lived as symbionts and ultimately gave rise to the nucleus” [8]. After Mereschkowsky’s death in 1921, Wilson included a scathing critique of this proposal in his 1928 textbook “*The Cell in Development and Heredity*”, following which there was little further discussion of this theory for nearly a generation [9]. It was not until 1970 that Margulis resurrected the notion of endosymbiosis, although her early work focused mainly on the mitochondria and chloroplasts, which had also fallen out of favor as having arisen in this fashion [10]. It took another eight years before the first archaeum was named as a potential ancestor of the cytoplasm and nucleus [11]. Shortly after this, Lake termed the concept of the nucleus as an endosymbiont the “endokaryotic” hypothesis, as opposed to a “karyogenic” one in which a nucleus developed (via an unspecified mechanism) in a protoeukaryote without the occurrence of an endosymbiotic event [12-14]. The former model has also been referred to as an endogenous, autogenous, or differentiation theory, while the latter is also known as the xenogenous theory [15]. Some are also careful to discriminate between endocytosis, involving a distinct host as well as a guest and the fusion of two equal prokaryotic partners to create a new cellular compartment [5,16]. These opposing eukaryogenic theories have been a hot topic of debate ever since. Unfortunately space does not allow for a full treatment of arguments in support of the karyogenic hypothesis, but some of the main concerns that have been raised will be addressed later in this paper [16-21]. The evidence which is put forth in support of one of these hypotheses or another is typically phylogenetic in nature. There are therefore two opposing types of phylogenetic trees which are supported by evidence: one in which eukaryotes arise from within the archaeal branches and which lends credence to various endosymbiotic or fusion theories and one in which eukaryotes remain equally separated from the prokaryotic domains (Figure 1) [22-26]. Confusingly, although there is consistently this dichotomy, there is no consensus on what the trees in question are called. The former has been called the Eocyte Tree [22,24], the Chimeric Model [23], the Two Primary Domains Scenario [25], and the Prokaryote/Eukaryote Dichotomy [26], while the latter has been referred to as either the Archaebacterial Tree/Model [22,23], the Three (Primary) Domains Tree/Scenario/Concept [24-26], or the Sister Groups Topology [27]. I have chosen to combine two of these naming schemes and will refer to this tree as the “Three Sisters Tree” (Figure 1A). Before delving into the phylogenetic data that exists, it is important to describe the current state of archaeal phylogenetics.

### Phylogeny of the archaea

**Figure 2:**
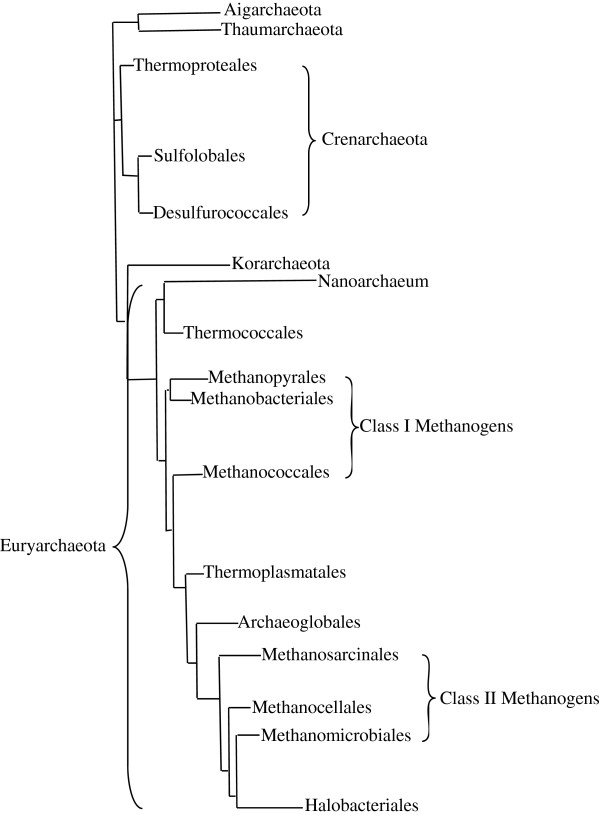
**Phylogeny of the Archaea based on ribosomal proteins.** Unrooted Bayesian tree of the archaeal domain based on the concatenation of 57 ribosomal proteins, including established phyla (−ota), major orders (−ales), as well as the two classes of methanogens. Figure based on reference 34.

For a half dozen years after the Archaea were elevated to domain status, along with Bacteria and Eukarya, they were imagined to consist of two distinct clades [28]. Woese and colleagues originally described two kingdoms (now called phyla) of archaea: Crenarchaeota (thermoacidophiles, sulfur-dependent bacteria, and extreme thermophiles) and Euryarchaeota (extreme halophiles, sulfate-reducing species, some thermophiles, as well as the methanogens) [28]. In 1996, a third phylum, Korarchaeota, was proposed based exclusively on environmental DNA sequences, then, following another six year period, it was proposed that Nanoarchaea deserved a phylum designation of its own [29,30]. While the former proposal has gone largely unchallenged, at least one group has argued that Nanoarchaea would be more accurately viewed as a deeply branching member of the Euryarchaeota [31-34]. Although there is currently no consensus concerning Nanoarchaeum’s precise phylogenetic position, I have chosen to use its inclusion in the Euryarchaeota as a working hypothesis in this paper. Six more years went by before a fourth phylum, the Thaumarchaeota, was separated from the traditional group of Crenarchaeota based on their formation of a separate phylogenetic clade as well as their propensity for mesophilic environments [35,36]. Recently, a fifth phylum was added to this mix: the Aigarchaeota, which, like the Korarchaeota, was based solely on environmental sequence data, and may represent a thermophilic relative of the Thaumarchaeota (Figure 2) [34,37,38]. Guy and Ettema have recently proposed a restoration of the two clade model by suggesting the existence of a “TACK” superphylum composed of all phyla described here with the exception of Euryarchaeota and Nanoarchaea. While recent analyses have actually grouped the Korarchaeota with the Euryarchaeota clade, they have not been implicated in any models for eukaryogenesis and I will thus leave this debate for another forum (Figure 2) [34].

Hypotheses proposing a particular archaeum as either a host or a guest in a specific endosymbiotic scenario typically originate from either of two approaches: one using cell biology or one using phylogenetic data [27]. The former method has largely been used in support of Euryarchaeota being involved in symbioses, while the latter method primarily supports the Crenarchaeota.

### Euryarchaeota as host: *Thermoplasma*

In 1978, Searcy and colleagues used cell biological techniques to name the euryarchaeum *Thermoplasma acidophilum* the host cell which ultimately gave rise to both the nucleus and the cytoplasm [11]. Their reasoning included the following cell biological observations that *Thermoplasma* contained: 1) histones, 2) a simple cytoskeleton, 3) microaerophilic respiration, and 4) no cell wall [11,39-41]. The fact that *Thermoplasma* was also a thermophilic acidophilic sulfur-metabolizing microbe also fit nicely into Searcy’s later theory that mitochondria and chloroplasts originated from sulfur-based symbioses [41,42]. The concept of *Thermoplasma* as a host organism was also appealing to Margulis who, in 1984, abruptly abandoned her original proposal that the bacteria *Mycoplasma* served as a host in the serial symbioses which led to the mitochondria, flagella, and chloroplasts, respectively [10,15]. Margulis went on to embrace *Thermoplasma* as a host for over a quarter of a century, until her death in 2011 [43-46]. Apparently, Margulis found the presence of histones in *Thermoplasma* particularly appealing since, by 1984, *Mycoplasma* was known to share the latter three observations listed above with *Thermoplasma*[47,48]. Further evidence of Margulis’ motivation comes from the fact that there was no mention of histones in her 1970 book but that her 1986 book was rife with discussion concerning these proteins [10,49]. Additional cell biological features given in support of *Thermoplasma* include evidence of a calcium signaling pathway, presence of proteasomes, and surface protein modification via N-glycosylation [50]. Phylogenetic support for Searcy’s choice of host comes from the use of supertree-based phylogenetic signal stripping to confirm that eukaryotic genomes nested within the archaeal ones examined, specifically as a sister group to the Thermoplasmatales [51]. It is interesting to note, however, that using a slightly different supertree method (a neighbor-joining distance based one opposed to one employing parsimony techniques), the authors obtained almost identical bootstrap values when the Thermoplasmatales were combined with the Nanoarchaea [51].

### Euryarchaeota as host: methanogens

Twenty years after *Thermoplasma* was proposed to be the host organism, Martin and Müller, using cell biological observations, proposed that the host was instead a hydrogen-dependent, anaerobic, strictly autotrophic euryarchaeum, namely one of the methanogens [6]. Their “hydrogen hypothesis” postulated that the α-proteobacterial ancestor of both hydrogenosomes as well as mitochondria gave off H_2_ as a waste product and that the methanogenic host became dependent on this wellspring of H_2_ once it was removed from a geological source [6]. Additional reasons for targeting methanogens other than those listed above include the fact that methanogenesis appears to be an ancient trait, that all three waste products (H_2_, H_2_O, and CO_2_) produced by anaerobic metabolism can be utilized by them, and that symbiotic association between this group and modern hydrogenosomes has been observed in nature [6]. The same month the hydrogen hypothesis was put forth, a similar but distinctive “syntrophy hypothesis” was proposed by Moreira and López-García [52]. One unique feature of this hypothesis is that it imagined two separate bacteria in a consortial relationship with a methanogen that blurred the lines of who actually played host and who played guest [53]. The first to partner with the methanogen in this scenario were sulfate-reducing myxobacteria belonging to the γ-proteobacterial group, these purportedly entered into a metabolic relationship much like that described in the hydrogen hypothesis [53]. It was then somewhat later that the mitochondrial ancestors may have joined the consortium, eventually adapting it to aerobic environments [53]. The authors of this hypothesis were critical of the use of *Thermoplasma* as a model host, claiming that its histones were more akin to bacterial HU proteins and that it contained gyrase, an enzyme which is lacking in certain methanogens as well as in all eukaryotes [53,54]. Despite these two convincing metabolism-based hypotheses, there has been no known study directly linking methanogens to eukaryotes using phylogenetic methods.

### Crenarchaeota as host

The first phylogenetic data to link eukaryotes with the Crenarchaeota consisted of morphological, not molecular sequence, data. In 1982, Lake and colleagues noticed that archaeal ribosome structure more closely resembled those from eukaryotes, and had soon proposed the “Eocyte Tree” based on this relationship (Figure 1B) [12-14]. The name of this tree is somewhat unfortunate, since the term eocyte, being another name for Crenarchaeota, ignores any phylogenetic data linking Euryarchaeota to eukaryotes (Figure 1C) [51,55]. Case in point, when Zillig and colleagues published their own phylogenies based on RNA polymerase morphology, they grouped eukaryotes with the eocytes, but freely admitted that, based on their data, the branching point for eukaryotes could equally be placed directly between the two phyla [56]. I have attempted to remedy this by renaming the Eocyte Tree the “Crenote” Tree, a term suggested by Woese as an informal name for Crenarcheaota (Figure 1B) [28]. In addition, the tree linking eukaryotes more closely with the Euryarchaeota has been labeled the “Euryote” Tree, another term suggested by Woese (Figure 1C) [28]. Interestingly, neither of these terms have garnered wide acceptance, as they have appeared in only a handful of publications over the score of intervening years since they were suggested. By 1987, Lake began including molecular sequence data in his analyses, upholding the Eocyte Tree by comparing 16 S rRNA sequences from four bacteria, four eukaryotes, and one crenarchaea: *Thermoproteus*, the closest eocyte to eukaryotes in Zillig’s tree [56,57]. One of the problems with identifying a putative crenarchaeal host using phylogenetics is that the final candidate ultimately depends on which sequences are chosen for analyses. Zillig had focused on the “sulfur-dependent” eocytes *Thermoproteus* and *Sulfolobus* in his work and these two genera became favored in most future work by others [56,58]. *Sulfolobus*, for instance, was linked to eukaryotes through the use of signature sequences in a H^+^-depending A1AO ATP synthase [59]. Signature sequences are conserved insertions or deletions in molecular sequences which are restricted to particular taxa [60]. Here, an 88-amino acid stretch was found to be present in both *Sulfolobus* and eukaryotes, but not in bacterial versions of H^+^-depending A1AO ATP synthase [59]. Further studies using signature sequences in the translational elongation factor EF-1α chose four eocytes for their study, two in the order Sulfolobales (including *Sulfolobus*), and two in the order Desulfurococcales [22]. An 11-amino acid sequence was found inserted into this protein in both the eukaryotes as well as the eocytes, with the Desulfurococcales sequence actually being closer to the eukaryotic consensus sequence [22]. Both composite as well as combined trees confirmed this close relationship between eukaryotes and Desulfurococcales using the sequences from both EF-1α and EF-2, although these techniques also introduced some ambiguity about whether Euryarchaeota or Crenarchaeota were more closely related to eukaryotes [61,62]. Additional ESPs (eukaryotic signature proteins) found in Crenarchaeota include RNA polymerase subunits RPB8 and RPC34 [63]. Further phylogenetic support for crenarchaeal hosts came from the analysis of 45 concatenated proteins which spanned the three domains, the most highly supported phylogenetic tree of which grouped eukaryotes, representatives of the three crenarchaeal orders (Thermoproteales, Sulfolobales, and Desulfurococcales), as well as *Nanoarchaeum* into a single clade [24].

### Thaumarchaeota as guest

Forterre proposed the most recent novel hypothesis for the archaeal origin of the nucleus in 2011 [26]. The hypothesis had its origin when he and his collaborators noticed that DNA topoisomerase IB, a protein common to eukaryotes but previously unknown to archaea, was found in certain “mesophilic crenarchaea”, the newly established phyla of Thaumarchaeota [64]. As in the euryarchaeal hypotheses, cell biological techniques were used to link Thaumarchaeota with the eukaryotes; one major difference with other hypotheses is that the archaeum was envisioned as a guest in this scenario with a bacterium acting as the host [26]. In addition to the presence of Topo IB, the other features that Thaumarchaeota were noted to share with eukaryotes were a large RNA polymerase A subunit that was not split in two, as well as two types of SSB, single stranded DNA binding proteins [26]. The presence of histones in Thaumarchaeota is of particular interest since this class of proteins has been found to be absent in the thermophilic Crenarchaeota [35]. The host in Forterre’s hypothesis consisted of a bacterium belonging to the PVC, Planctomyces-Verrucomicrobia-Chlamydia superphylum [25,26]. The choice of a PVC bacterium is closely tied to aspects of its membrane as well as its cytoskeletal elements [26]. Particularly of interest is that members of the PVC phylum are (proposed and) discussed to have the following features: 1) contain analogs of eukaryotic membrane coat proteins such as clathrin and nucleoporin, 2) exhibit an intracytoplasmic membrane that surrounds the bacterial nucleoid, 3) demonstrate sterol biosynthesis, 4) have been known to have large cells that divide by budding, 5) contain homologs of α- and β-tubulin in place of FtsZ, 6) have apparently developed a simple form of endocytosis, despite the fact that all modern PVC have a cell envelope and therefore cannot perform phagocytosis [26]. Phylogenetic support for Thaumarchaeota acting as guests in this scenario comes from the analysis of 3537 orthologous protein sequences and the determination of the intersection of this dataset with eukaryotic sequence [65]. The top three most likely candidates to lie at the intersection position include two Thaumarchaeota (*Cenarchaeum* and *Nitrosopumilus*) and *Nanoarchaeum*, respectively [65].

### The impasse

Phylogenetic data discussed above have been used in support of Euryarchaeaota, Crenarchaeaota, as well as Thaumarchaeaota as either a host or a guest in the endosymbiotic origin of the nucleus [24,51,61,62,65]. Obviously, it is rather unlikely that all these occurred, and that the resulting chimeras finally succeeded to survive and to evolve, until today. In addition to the phylogenetic data presented already, Shinozawa and colleagues have linked yeast ORFs (open reading frames) with archaea using a technique called homology-hit analysis [66,67]. Although evidence was presented linking eukaryotic nuclear genes to archaea, no specific phyla of archaea were initially indicated [66,67]. After refining their techniques, as well as adding three additional eukaryotes (fission yeast, fruit fly, and roundworm) to their dataset, these researchers named *Pyrococcus*, a euryarchaeum, as the probable host species [55]. A few years after this, however, by including human and *Arabidopsis* ORFs in their analysis in place of the four eukaryotes listed above, Shinozawa’s lab backed off their identification of *Pyrococcus* and concluded that the nuclear genome descended from uncharacterized archaea which did not belong to any of the major phyla [68]. This latter conclusion was also reached by Koonin and colleagues, who analyzed 355 eukaryotic genes of putative archaeal origin and concluded that their descent was from a distinct, ancient, but uncharacterized archaeum [69]. The lack of agreement by many large-scale phylogenetic studies led a group of researchers to subtitle a recent paper “are we at a phylogenetic impasse?” [25]. A few years before this, Embley and Martin had put the same problem slightly differently, “Although individual analyses of informational genes arrive at fundamentally different interpretations, no one has yet suggested that more than one archaebacterium participated in eukaryote origins” [70]. While it is uncertain whether this statement was meant as a simple musing or, rather, as a challenge- it seems to be an idea whose time has come.

### *Ignicoccus* as a crenarchaeal host

Huber and Rachel, along with their colleagues, in a paper describing properties of the hyperthermophilic, strictly anaerobic, chemolithoautotrophic, sulfur-reducing crenarchaeum *Ignicoccus hospitalis*, recently put forth what they described as an “extreme hypothesis” [71]. “Would not an organism like *I. hospitalis*, with its huge and energized periplasmic space, be an ideal candidate for such an ancestor (of eukaryotes) …?” they speculated [71]. Far from being an extreme hypothesis, I would like to suggest that *I. hospitalis*, acting as an obligate host for its ectosymbiont/ectoparasite ‘*Nanoarchaeum equitans’*, solves the phylogenetic impasse described above. Huber and Rachel have been studying *Ignicoccus* for the last dozen years since they described the first two species that had been isolated from samples taken at submarine hydrothermal vents in the Atlantic Ocean, north of Iceland, as well as in the Pacific Ocean [72]. This genus is of particular interest in that it is the only archaeum besides *Thermoplasma* to lack a cell wall [73]. In addition to this, *Ignicoccus* is the only archaeum to exhibit two membranes, an outer cellular membrane, or OCM, that had been compared to that of gram-negative bacteria, and an inner (cytoplasmic) membrane [74,75]. This double membrane system has recently been compared to that of *Plantomycetes*, one member of the PVC group discussed above [71]. The OCM is unique in that it contains diether lipids but not tetraether ones, and it does not contain any beta-barrel porins or LPS, compared to gram-negative bacteria [74]. The intermembrane compartment (formerly referred to as the periplasmic space), is the largest feature of the cell, with a volume that is 2–3 times greater than that of the cytoplasm itself [71,74]. Another feature that *Ignicoccus* apparently shares with the PVC group of bacteria is the existence of a simple form of endocytosis, but in this case *Ignicoccus* appears to demonstrate exocytosis as well [74]. Numerous vesicles can typically be seen in the periplasm, and sometimes vesicles can be seen on the outer cell surface, either in a release or a fusion process [76]. Extracellular vesicles of this nature have also been observed in some gram negative bacteria [76]. In electron micrographs, the inner membrane appears undulating and vesicles have been seen to bleb off in either direction from it [77]. *Ignicoccus* is therefore the first archaeum that is likely to possess a mechanism for lipid translocation and sorting [77]. Although it is not presently known what proteins are involved in vesicle formation it is note-worthy that *Ignicoccus* has genes encoding all three Cdv (cell division) proteins: A, B, and C, the latter two of which display homology to the eukaryotic ESCT-III sorting complex that has been linked to the budding of luminal vesicles [78]. In addition to vesicular transport, *Ignicoccus* has been seen to exhibit a simple cytoskeleton [73]. Straight, or slightly bent, longitudinal fibers, up to 500 nm long and 300 nm wide were observed in the cytoplasm, as were what appeared to be oblique sections of putative cytoskeletal bundles [73]. Multiple circular pores have also been visualized dotting the OCM which have been compared with those created by porin proteins in gram negative bacteria [77]. The use of *Ignicoccus* as a host in an endosymbiosis/fusion scenario would explain much of the phylogenetic data that has been generated which links Crenarchaeota to eukaryotes. *Ignicoccus*, for instance, belongs to the order Desulfurococcales and therefore contains the EF-1α signature sequence described above. In summary, *Ignicoccus* resembles *Thermoplasma* in its lack of a cell wall, has the metabolism described in the syntrophy hypothesis (it reduces elemental sulfur to H_2_S), explains the generation of a Crenote Tree, and shares most of the features with PVC bacteria which make them a convincing host candidate. These facts alone may be convincing enough to promote Huber and Rachel’s “extreme hypothesis” but the other half of the puzzle lies with *I. hospitalis*’ ectosymbiont/ectoparasite- *Nanoarchaeum*.

### Nanoarchaeum as a euryarchaeal host/guest

*‘Nanoarchaeum equitans’* was discovered a couple of years after *Ignicoccus* since the first two species described lacked ectosymbionts [30]. *I. hospitalis*, however, was found to live in close association with tiny spherical *N. equitans* which were about 400 nm in diameter and which, at 0.5 megabases, contained the smallest known archaeal genome [30]. During exponential phase in *Ignicoccus*, the numbers of *N. equitans* are at their minimum, occupying only 25% of cells with numbers per cell ranging between one and three, but during stationary phase up to 90% of cells are seen to be occupied by approximately 10 *N. equitans*[79]. The greatly reduced genome of *N. equitans*, along with molecular features such as the presence of split-genes and the absence of operons support the belief that it represents a very ancient line of the archaea [80-83]. The *N. equitans* genome mainly encodes the machinery involved in informational processing and repair, but lacks the genes required to manufacture lipids, cofactors, amino acids, and nucleotides [84]. Because of this, there are few cell biological features that *N. equitans* does not share with *Ignicoccus* which need to be described here, sharing as it does the same membrane components and metabolism described above. One exception includes the presence of the tubulin homolog FtsZ, a protein that is lacking in crenarchaea [73,84,85]. Another exception is the presence of histones, a class of protein which is lacking in crenarchaea and one of the reasons given for supporting Thaumarchaeota as a host [35]. In fact, *N. equitans* is the only archaeum to contain a *bone fide* homolog of histone H3, as defined by having a signature sequence consisting of a lysine-containing oligopeptide inserted into the L1 loop region between the two α-helices of the histone fold [86]. This is significant since all other archaeal histones can, by this definition, be considered homologs of histone H4 [87]. What is more, the H3 homolog of *Nanoarchaeum* was shown to be unable to compact DNA in the absence of the *N. equitans* H4 homolog, hinting at a system more similar to that used in eukaryotes [86]. Eukaryotes are known to form only H3/H4 heterotetramers whereas all archaea with the exception of *N. equitans* appear to form both homo- and heterodimers of two of the same or two different histone H4 subtypes, respectively, which then bind DNA as tetramers [88]. In addition to the cell biological evidence, *N. equitans* has been indicated in two separate phylogenetic investigations, albeit both times not as the primary genus identified, but rather as an alternate viable candidate [51,65]. Alone, perhaps, the phylogenetic data linking either *Ignicoccus* or *Nanoarchaeum* to eukaryotes is not particularly convincing, but combining a euryarchaeal genome with a crenarchaeal one explains much of the data described above and allows us to break through the phylogenetic impasse. For this reason, I have proposed the Synote Tree as a replacement for both the Crenote and the Euryote Trees (Figure 1D). Assuming from the phylogenetic data that at some time in the past the two archaea involved fused their genetic material, there are three possibilities concerning this event: 1) nanoarchaeal genes were transferred into the cytoplasm of *Ignicoccus* before an α-proteobacteria became an endosymbiont, 2) the entire nanoarchaeum was transferred into the *Ignicoccus* proto-nucleus upon the arrival of the α-proteobacteria, or 3) the nanoarchaeal genes were transferred into the nucleus of the proto-eukaryote at a later date. The first possibility seems unlikely since the genome of *N. equitans* is considered to be evolutionarily stable compared with many bacterial parasites [84]. Although a prior fusion with the *Ignicoccus* genome cannot be completely ruled out, since this event is not known to have ever taken place again during the intervening years, the chance of an α-proteobacterium merging with what must be, at best, an exceedingly rare occurrence seems very low. The second possibility also has some difficulties, perhaps not in a physical sense since, during stationary phase the *Ignicoccus* are dotted with *N. equitans* which may have been internalized by a bacterial endosymbiotic event, but more in a mechanistic sense in that two cases of endosymbiosis would had to have been established almost simultaneously [79]. This middle possibility does have a certain degree of attractiveness, however, in that it could explain the topography of the nucleus if the former cytoplasm of the *Ignicoccus* collapsed down upon the invading *Nanoarchaeum*, creating a spherical space surrounded by a contiguous and highly invaginated membrane resembling the modern endoplasmic reticulum. This last prediction comes from the fact that more than 80% of *N. equitans* are found attached to *Ignicoccus* at sites where the outer and inner membranes appear to be in direct contact, suggesting that the inner membrane is attracted to the ectosymbiont/ectoparasite in some way [73]. Another extremely “nuclear” feature that *Nanoarchaea* were found to have was their ability to inhibit the proliferation of their host cells, perhaps preventing *Ignicoccus* from dividing until they have had a chance to divide [79]. The transfer of nanoarchaeal genes to *Ignicoccus* following the bacterial endosymbiotic event is probably the most likely of the three possibilities presented above. Perhaps some adaptation to having an α-proteobacterial endosymbiont accelerated the transfer of genes between single or multiple ectosymbionts and the nucleus. Regardless of when this transfer/fusion happened- exactly how could a pair of archaea have served as a host to the bacterial endosymbiont which later became the mitochondrion?

### The extreme hypothesis

The time: a couple of billion years ago, the place: a submarine hydrothermal vent, the players: the ancestors of *Ignicoccus*, *Nanoarchaeum*, as well as the α-proteobacterial precursor of the mitochondrion. Others have undertaken explanations of how life itself likely got its start at this very location [89,90]. Recent studies have demonstrated that archaea such as *Ignicoccus* and *N. equitans* still live in close association with α-proteobacteria in such areas [91,92]. This latter group would be expected to be thermo-tolerant to some degree in order to establish an endosymbiotic relationship with the pair of thermophilic archaea. It is interesting that almost all thermophilic forms of α-proteobacteria characterized to date are in the order Rhizobiales, a not-so-distant relative of the Rickettsiales from which mitochondria are presumably derived [93-97]. This is, then, in the quite literal sense, an extreme hypothesis, since all the participants lived at high temperatures. That is not to say that there didn’t have to be a considerable amount of compromise to make this arrangement work. Current thermophilic α-proteobacteria have an optimum growth temperature around 50°C, whereas *Ignicoccus* does not typically grow below 75°C [93-98]. *N. equitans*, for its part, has a G-C content of only 31.6%, compared to *Ignicoccus’* 56.5% and thus has a melting temperature well below that of *Ignicoccus’* optimum temperature, making it a rather poor thermophile in the first place [99]. The arrival of an α-proteobacterial endosymbiont may have been linked with the breakage of a venting chimney spire which may have then eased the new consortium into a lower growing temperature than the archaea had been previously used to [100]. *Ignicoccus* has an interesting response to being grown at sub-optimal temperatures- it shuts down the biosynthetic processes leading to tetraether formation, thus changing the makeup of its inner membrane [98]. Martin has hypothesized that the bacterial membrane synthesis pathway eventually replaced that of its archaeal partners and points out that an archaeal-derived isoprene synthesis pathway still exists in eukaryotes but has been adapted for the synthesis of sterols, quinone tails, and dolichol phosphate instead of lipids *per se*[101]. Concerning membranes, opponents of endosymbiotic theories claim that phagocytosis must be a prerequisite for internalizing other microbes, something that has not been seen in any prokaryote to date [19,20]. Others, however, attest that a tear in a membrane may have opened up the host to invasion [102,103]. Electron microscopic studies have actually revealed defects in both *Ignicoccus* membranes in the form of fractures appearing in non-contiguous membranes, although it is not clear whether this was an artifact of preparation or not [73,74]. In addition to this, *Ignicoccus* was recently shown to have cell surface appendages which may act in adhesion [104]. *Ignicoccus* may thus have inadvertently captured an α-proteobacteria as cooler seawater rushed in to a broken chimney spire and then engulfed it via a nearby tear in the outer cellular membrane. Regardless of the precise mechanism, once the bacterium was engulfed, it effectively created two very different compartments within the archaeal host. The former cytoplasm contained within the inner membrane now effectively became the nucleus and the former massive periplasm now became the new cytoplasm. It was this partitioning effect that first piqued the interest of Rachel and Huber when they proposed that *Ignicoccus* would make a good host [71]. This partitioning led to another interesting effect- archaeal 80 S ribosomes were now contained within the nucleus, requiring an export of either themselves or at least their protein products into the nascent cytoplasm. Luckily, the inner (now effectively the nuclear) membrane was able to bud vesicles out into the new cytoplasm [78]. This arrangement could explain the problem of which came first, the nucleus or the nuclear pores. Some have pointed out that the former is useless without the latter as well as vice versa [105]. From this model, it would seem that the nucleus initially achieved import and export by vesicle budding, a role that was later delegated to the ER when the nuclear pores developed. It has recently been discussed that coated vesicles and nuclear pore complexes might share a common evolutionary origin [105-107]. The initial need to export ribosomes into the cytoplasm may explain the somewhat convoluted system which still exists in eukaryotes today- the components of ribosomes are encoded for in the nucleus, the mRNA for ribosomal proteins is then exported into the cytoplasm, translated, and the resulting proteins are imported back into the nucleus for ribosome assembly, followed by the final export of ribosomes back into the cytoplasm [107,108].

## Conclusions

Although certainly not the first model for eukaryogenesis, an endosymbiotic event which involved a proto-mitochondrial α-proteobacterium and a pair of closely associated archaea (ancestors of *Ignicoccus* and *Nanoarchaeum*) would break through an impasse that has been reached in research into the origins of eukaryotes by creating the Synote Tree. In addition to the cell biological queries for which it offers a solution, there are, admittedly, a number of further problems that must be solved if this latest theory is to survive under scrutiny. If it does, it is nothing short of amazing how apt Mereschkowsky’s 1905 description of small micrococci invading a bacterium and living as symbionts before eventually becoming the nucleus turned out to be [8].

## Competing interests

The author declares that he has no competing interests.

## Author’s contributions

JG performed all of the work associated with this manuscript. JG conceived of the study, carried out the analyses, formulated the hypotheses, and drafted the manuscript. The author had read and approved the final manuscript.
